# Supplementary material for: Direct Photochemical Synthesis of Substituted Benzo[b]fluorenes
Source: Org Lett. 2024 Nov 25;26(48):10364–8. doi: 10.1021/acs.orglett.4c03978 (PMC11629382; doi:10.1021/acs.orglett.4c03978)
Supplement: Supplementary file 2 — ol4c03978_si_002.zip [file ol4c03978_si_002.zip › FID for Publication/5s/20240724_RC-2-C-5-132A_01/plots/RC-2-C-5-132A_HSQCAD_20240724_01_plot01.pdf]

RC-2-C-5-132A

Location 4 | Pulse sequence HSQCAD | Temperature 25 | Suboperator RuairiCrawford
Date collected 2024-07-24 | Solvent acetone | Spectrometer localhost.localdomain-vnmrs500 | Operator MBaumann

SAMPLE

date Jul 24 2024
solvent acetone
sample 20240724_RC-2-C-5-132A_01

ACQUISITION

sw 4595.6
at 0.150
np 1378
fb 4000
ss 32
d1 1.000
nt 2

2D ACQUISITION

sw1 21361.8
ni 96
phase arrayed

PRESATURATION

satmode n
wet n

TRANSMITTER

tn H1
sfrq 499.724
tof -206.6
tpwr 58
pw 6.300

DECOUPLER

dn C13
dof -2485.2
dm nny
decwave W40_P7374
dmf 32258
dpwr 43
pwxlvl 62
pwx 14.000

HSQC

j1xh 146.0
nullflg y
mult 2

FLAGS

hs nn
sspul y
PFGflg y
hsglvl 5736

ADIABATIC

pwx180ad P7374_ad300
pwx180adR P7374_ad300R
pwx180 465.4
pwxlvl180 57
pwx180ref P7374_ref200
pwx180r 2000.2
pwxlvl180r 48

SPECIAL

temp 25.0
gain 34
spin 0

F2 PROCESSING

gf 0.069
gfs not used
fn 2048

F1 PROCESSING

gf1 0.004
gfs1 not used
proc1 lp
fn1 2048

DISPLAY

sp -5.7
wp 4595.6
sp1 -1257.4
wp1 21361.8
rfl 5.7
rfp 0
rfl1 1257.4
rfp1 0

PLOT

wc 142.6
sc 0
wc2 127.1
sc2 12.7
vs 128
th 2
ai cdc ph

ACQUISITION ARRAYS

array phase
arraydim 192
i phase
1 1
2 2

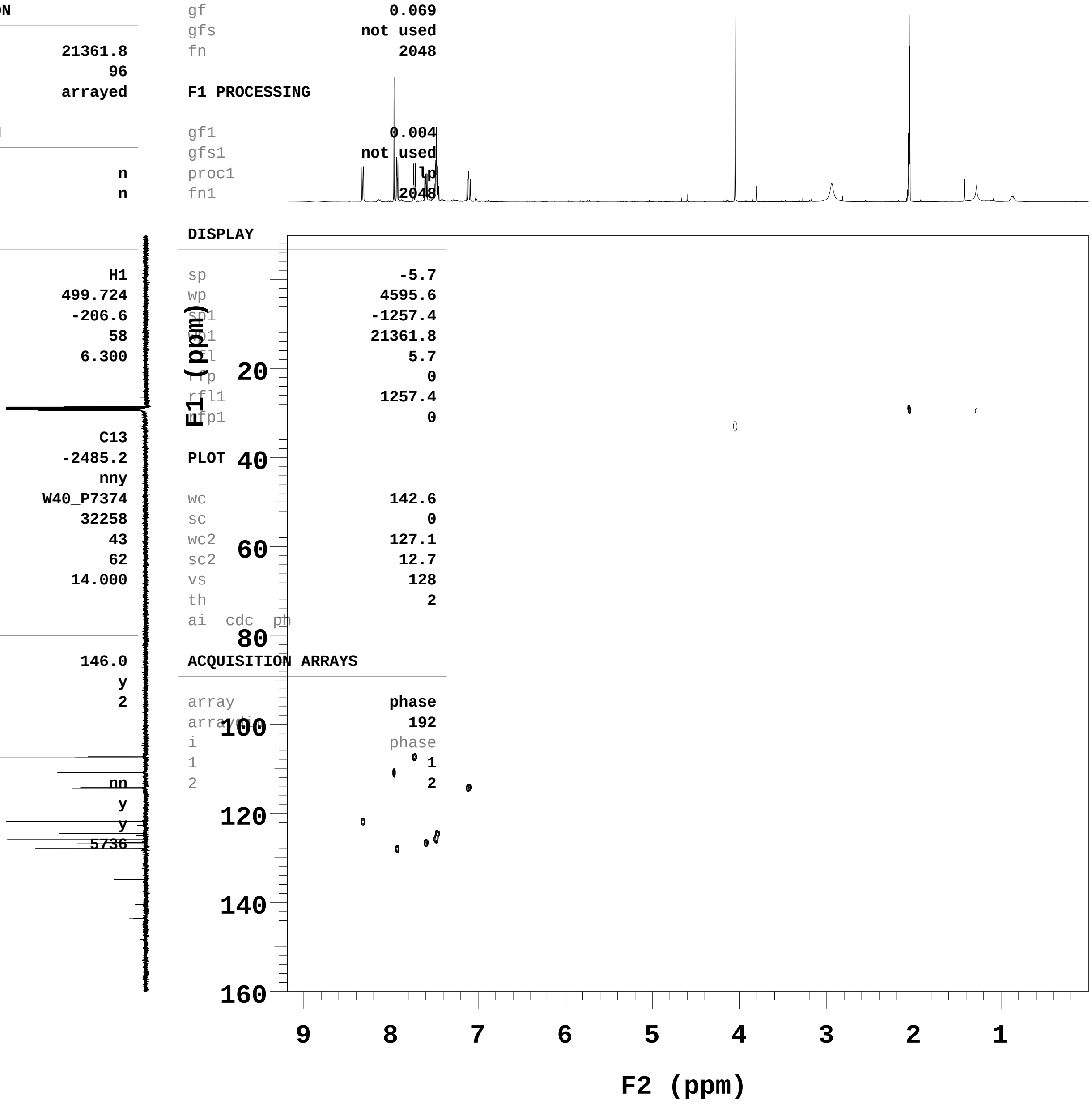

Plotname: RC-2-C-5-132A_HSQCAD_20240724_01_plot01

Data file /home/data/MBaumann/cscb500/RuairiCrawford/2024/20240724_RC-2-C-5-132A_01/RC-2-C-5-132A_HSQCAD_20240724_01.fid Plot date 2024-07-24
